# Applications of 3D Printing and Artificial Intelligence in Healthcare Management: A Narrative Review

**DOI:** 10.3390/bioengineering13020196

**Published:** 2026-02-09

**Authors:** Conrado Domínguez Trujillo, Donato Monopoli Forleo, Carmen Delia Dávila Quintana, Juan Mora Delgado

**Affiliations:** 1 Canary Islands Health Service, University of Las Palmas de Gran Canaria, 35001 Las Palmas de Gran Canaria, Spain; 2 Department of Biomedical Engineering, Instituto Tecnológico de Canarias, 35003 Las Palmas de Gran Canaria, Spain; 3 Departamento de Métodos Cuantitativos en Economía y Gestión, University of Las Palmas de Gran Canaria, 35017 Las Palmas de Gran Canaria, Spain; 4 GICAMI (Grupo Investigación Clínico Asistencial de Medicina Interna) Group, Infectious Diseases and Microbiology Unit, Instituto de Investigación e Innovación Biomédica de Cádiz (INiBICA), Hospital Universitario de Jerez de la Frontera, 11407 Jerez de la Frontera, Spain

**Keywords:** printing, three-dimensional, artificial intelligence, bioprinting, biomedical engineering, patient-specific modeling, machine learning

## Abstract

The integration of 3D printing and artificial intelligence is transforming healthcare management by driving innovations in personalized care, supply chain operations, and clinical workflows. This review offers a comprehensive overview and in-depth analysis of recent (2018–2025) applications where AI technologies enhance 3D printing within healthcare. We explore how AI-powered design and optimization facilitate the creation of patient-specific medical devices, implants, and even bioprinted tissues, while intelligent process control increases both quality and efficiency. Additionally, we examine regulatory and ethical considerations, including the evolution of frameworks for AI-enabled devices, as well as challenges in data governance, validation, and equitable access. The review takes a global perspective, presenting real-world case studies that showcase both successful implementations and ongoing challenges. We also discuss various perspectives and controversies, such as the balance between innovation and safety in autonomous AI design, and highlight areas where further research is needed. In contrast to previous narrative reviews that focus solely on clinical applications or technical aspects, this review uniquely evaluates the combined impact of AI and 3D printing on healthcare management—including cost-effectiveness, governance, decision-making processes, and point-of-care manufacturing. This work is particularly valuable for hospital administrators, clinical operations leaders, health policymakers, and biomedical innovation teams seeking to understand the broader implications of AI-enhanced 3D printing in healthcare management. Nevertheless, despite promising advancements, the field is constrained by heterogeneous evidence, a lack of standardized evaluation metrics, and insufficient long-term outcome data, which together limit the ability to fully assess the sustained impact of AI-integrated 3D printing in healthcare environments.

## 1. Introduction

Additive manufacturing, commonly known as 3D printing, has emerged as a transformative technology in healthcare, enabling the fabrication of complex and patient-specific medical products on demand [1]. From anatomical models for surgical planning [2] to customized implants and prosthetics [3], 3D printing allows healthcare providers to create bespoke solutions tailored to individual patient anatomies and needs.

Its advantages over traditional manufacturing include design flexibility, rapid prototyping, and the ability to produce one-off items cost-effectively without mass production [4]. Over the last decade, hospitals and clinics worldwide have begun adopting 3D printing, with hundreds of institutions establishing in-house fabrication labs [5].

Concurrently, artificial intelligence has rapidly advanced and found myriad applications in healthcare. AI techniques such as machine learning (ML) and deep learning can discover patterns in large datasets and optimize complex processes, while natural language processing and intelligent robotics are improving decision support and automation [6]. Recent studies highlight that incorporating AI into 3D printing enables new capabilities, including reconstructing patient-specific models from medical images, selecting and optimizing print materials and parameters, and monitoring products across their life cycle [7,8,9].

In essence, artificial intelligence serves as a catalyst that enhances the precision and versatility of 3D printing, enabling dynamic optimization and highly individualized solutions that surpass the capabilities of traditional methods [10,11].

The objectives of this review are to:

1. Summarize recent developments (2018–2025) in AI-enhanced 3D printing relevant to healthcare systems;
2. Analyze their implications for management domains such as cost, workflow, and regulation;
3. Identify implementation models and challenges in different healthcare settings.

This review aims to support a diverse audience, including hospital managers, clinicians, biomedical engineers, and policymakers, by synthesizing cross-disciplinary insights into the operational and strategic integration of AI and 3D printing in healthcare. While technological advancements in AI and 3D printing underpin this review, the primary focus is on their implications for healthcare management. This includes examining how these technologies impact decision-making processes, cost-efficiency, workflow integration, and governance within healthcare systems.

### Literature Search Strategy

This study follows a narrative review approach rather than a systematic review. Narrative reviews are particularly suited to emerging, interdisciplinary fields, such as the convergence of AI and 3D printing, where evidence is heterogeneous, methodologies vary widely, and conceptual integration is required. Unlike systematic reviews, which use predefined protocols, exhaustive search strategies, and formal risk-of-bias assessments, narrative reviews aim to provide a broad interpretative synthesis of trends, implementation models, and managerial implications. The thematic synthesis used here is interpretative rather than quantitative, enabling integration of clinical, operational, economic, and regulatory perspectives from diverse sources.

A non-systematic search strategy was employed to identify relevant peer-reviewed publications, reports, and case studies. Literature was retrieved from electronic databases including PubMed, Scopus, and Google Scholar, using combinations of keywords and MeSH terms such as “3D Printing”, “Artificial Intelligence”, “Healthcare Management”, “Bioprinting”, “Machine Learning”, “Personalized Medicine”, and “Medical Device Manufacturing”.

We included peer-reviewed articles, reviews, reports, and case studies published between 2018 and 2025 that explicitly addressed the integration of 3D printing and AI within healthcare management domains. Exclusion criteria included articles focused solely on technical development without healthcare context. Out of 372 records identified through database and manual searches, 94 were included after screening for relevance.

Thematic synthesis was used to organize the evidence across four managerial domains: (1) clinical value, (2) operational efficiency, (3) economic impact, and (4) governance and ethics. These themes provided a framework for structuring the review and ensuring consistency in synthesis.

**Figure 1:**
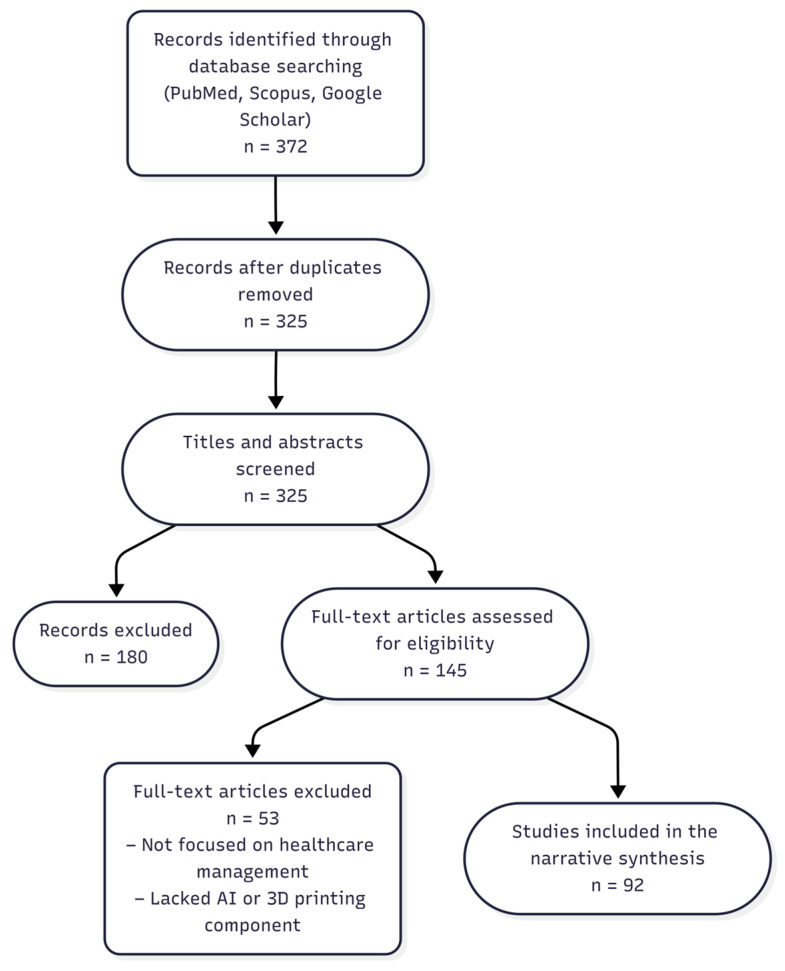
Simplified flow diagram summarizing the literature selection process.

A simplified flow diagram summarizing the literature selection process has been added (Figure 1). The selected time range (2018–2025) captures the most recent and relevant developments, particularly post-pandemic innovations and regulatory shifts. Given the exploratory nature of many implementations and limited longitudinal data, caution is warranted when generalizing reported outcomes.

## 2. Technological Foundations and Current State

### 2.1. Fundamental Principles of 3D Printing in Healthcare

Three-dimensional printing, also known as additive manufacturing (AM), has emerged as a revolutionary technology in healthcare, enabling the creation of complex, customized medical solutions that were previously impossible or prohibitively expensive [12].

The fundamental principle of 3D printing involves creating physical objects layer by layer from digital models, offering unprecedented flexibility in design and production. The technology enables healthcare providers to create patient-specific solutions that perfectly match individual anatomical structures, thereby improving treatment outcomes and patient satisfaction [13]. 3D printing encompasses various techniques including fused deposition modeling (FDM), stereolithography (SLA), selective laser sintering (SLS), PolyJet and bioprinting, each with specific applications in healthcare contexts. Despite its tremendous potential, literature reviews indicate that 3D printing remains in the initial phase of implementation in healthcare, with significant untapped potential awaiting exploration [14].

The adaptability of 3D printing is clear in its ability to use a wide range of materials, including polymers, metals, ceramics, and even living cells (known as bioinks) to create healthcare solutions. This material flexibility makes it possible to produce everything from rigid orthopedic implants to flexible anatomical models for surgical planning. Recent developments have expanded the selection of printable biomaterials, such as biodegradable polymers that can gradually be replaced by a patient’s own tissue, paving the way for new possibilities in regenerative medicine [15].

3D printing techniques are evolving rapidly, with each new innovation expanding the range of possible healthcare applications. For example, multi-material printing now enables the creation of objects with different physical properties in separate regions, closely replicating the complexity of biological tissues. Digital Light Processing (DLP) technology offers faster printing speeds and higher resolution, which are essential for producing detailed anatomical models [16].

The evolution of bioprinting includes the development of 4D bioprinting, where printed constructs can change their shape or function over time in response to stimuli like temperature or chemical gradients. These technological foundations underpin the transformative potential of 3D printing in healthcare management, enabling a shift toward more personalized, efficient, and effective patient care models [17].

### 2.2. Artificial Intelligence Integration in Healthcare Manufacturing

Artificial intelligence has become increasingly integral to healthcare management, offering powerful capabilities for data analysis, decision support, and process optimization (Table 1). When integrated with 3D printing technologies, AI enhances every stage of the manufacturing process, from design conceptualization to production monitoring and quality control. Machine learning algorithms can analyze patient imaging data to automatically generate optimized 3D models for printing, reducing the time-intensive manual segmentation process traditionally performed by skilled technicians. This automation not only accelerates production but also improves consistency and reduces human error, critical factors in medical applications where precision is paramount [18,19].

Deep learning neural networks have revolutionized medical image processing, enabling automatic recognition and segmentation of anatomical structures from CT, MRI, and ultrasound scans. These AI systems can identify pathological features and normal structures with accuracy rivaling human experts, but with greater consistency and speed [20]. The AI-generated models can then be refined for 3D printing, creating anatomically accurate physical representations for presurgical planning, medical education, or implant design [21,22].

Beyond design optimization, AI systems contribute to process monitoring and quality assurance in medical 3D printing. Predictive analytics can anticipate material behavior during printing, adjusting parameters dynamically to ensure optimal results [23].

Furthermore, AI can optimize production scheduling and resource allocation in healthcare manufacturing settings, maximizing equipment utilization and minimizing waste. Reinforcement Learning (RL) is increasingly used to dynamically adjust printing parameters such as temperature, printing speed, and layer height to maintain optimal quality [24]. The synergy between AI and 3D printing creates an intelligent manufacturing ecosystem particularly valuable in healthcare contexts where personalization, precision, and quality control are essential requirements [10].

Algorithmic bias in deep learning-based image segmentation emerges from imbalanced datasets, scanner- and site-specific acquisition protocols, and human annotation practices, producing systematically worse performance for particular demographic, anatomical, or institutional subgroups [25].

### 2.3. Current Applications in Clinical Practice

The integration of 3D printing and AI has found numerous applications across diverse clinical specialties, with particularly notable impacts in orthopedics, maxillofacial surgery, cardiology, and oncology. In orthopedic settings, patient-specific surgical guides created through 3D printing have demonstrated improved precision in joint replacement procedures, reducing operating time and enhancing post-operative outcomes [26]. These technological interventions not only improve clinical outcomes but also enhance operational efficiency by reducing surgical complications and associated resource utilization [3,4,27].

Surgical planning represents one of the most established applications of 3D printing in healthcare, with substantial evidence supporting its value in complex cases. Studies have documented reduced operative time, decreased blood loss, and improved accuracy when using 3D printed guides and models [28,29]. Similarly, in cases of surface osteosarcoma, 3D printing technology has facilitated conservative surgical management approaches that preserve function while ensuring complete tumor removal, demonstrating how these technologies can support less invasive treatment strategies with better functional outcomes [29]. The role of AI in surgical planning extends beyond model creation to simulating surgical outcomes. AI algorithms can predict the biomechanical effects of different surgical interventions, assisting surgeons in selecting the optimal approach [30].

Beyond surgical applications, the combination of 3D printing and AI has transformed the management of chronic wounds, especially diabetic foot ulcers (DFUs). With advanced 3D printing techniques, it is now possible to produce personalized wound dressings that fit the unique contours of irregular wounds, improving both the wound environment and the effectiveness of drug delivery [31,32].

AI algorithms assess wound characteristics to identify the best dressing designs and material compositions, while also tracking the healing process to recommend adjustments to treatment protocols. Research is actively advancing smart wound dressings equipped with sensors, enabling AI to analyze real-time wound data and refine treatment strategies for better outcomes [33].

### 2.4. AI-Enhanced 3D Printing Applications in Healthcare

In AI is being leveraged to bolster 3D printing across a range of healthcare applications. Broadly, these applications can be grouped into a few key categories:

- Design Optimization and Personalization: AI algorithms (including generative design and deep learning models) assist in creating complex, patient-specific designs for implants, prosthetics, and anatomical models. By learning from imaging data or prior designs, AI can suggest optimal geometries or material distributions that improve fit and function. This is critical for personalized medicine, where each product must be tailored to an individual patient’s anatomy or condition resulting in enhanced comfort and better treatment outcomes [27,34].
- Process Control and Quality Assurance: Machine learning and reinforcement learning techniques are used to monitor 3D printing processes in real time and adjust parameters to ensure quality. For example, AI computer vision systems can detect printing defects (like warping or misalignment) and correct them on the fly, reducing errors and waste. Such intelligent control is especially important when producing medical devices, which require high precision and reliability [8].
- Healthcare Supply Chain Management: AI-driven analytics and predictive models help determine when and where to 3D-print medical supplies or devices, improving responsiveness. On-demand printing at or near the point of care, guided by AI demand forecasting, can reduce the need to stockpile inventory and shorten delivery times. This has been useful for quickly producing items like surgical instruments, custom surgical guides, or even personal protective equipment during crises [35].
- Clinical Decision Support and Simulation: In combination with 3D printing, AI can support clinical decisions by simulating outcomes. For instance, patient imaging data can be analyzed by deep learning models to identify the optimal surgical approach, and a 3D-printed model of the patient’s anatomy can then be created for rehearsal. AI can also predict how a 3D-printed implant might behave under physiological conditions, aiding clinicians in selecting or modifying designs for better safety and efficacy [36].
- Automation and Workflow Integration: Natural language processing and other AI tools can help integrate 3D printing into hospital information systems. For example, NLP could scan surgical schedules and physician notes to identify cases that might benefit from a 3D-printed model or device, automatically flagging them for the hospital’s 3D printing lab. Reinforcement learning-based scheduling algorithms might optimize printer utilization when multiple print jobs (for different patients or departments) are queued, prioritizing urgent needs and minimizing idle time [37,38].

## 3. Healthcare Management Implications

### 3.1. Economic Considerations and Value Assessment

**Figure 2:**
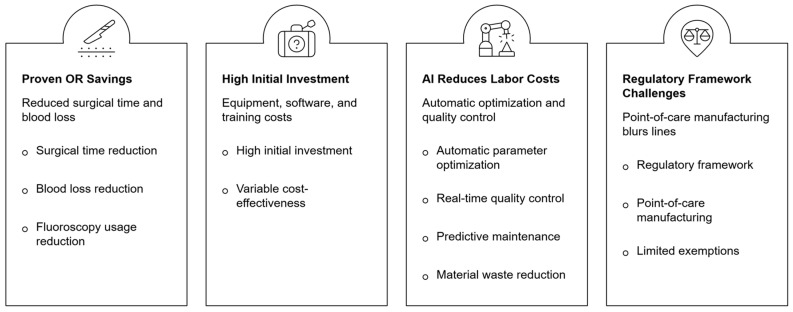
Key benefits, costs, operational impacts, and regulatory challenges of artificial intelligence implementation in surgical workflows and point-of-care manufacturing.

The economic impact of incorporating 3D printing and AI technologies into healthcare is multifaceted, involving substantial upfront investments, ongoing operational costs, and the potential for significant long-term savings (Figure 2). Setting up an in-house 3D printing lab requires major capital outlays for equipment, software, and specialized staff training. However, evidence from orthopedic departments shows that these investments can be cost-effective when evaluated holistically. Conducting thorough Total Cost of Ownership (TCO) analyses is essential to accurately assess the long-term economic benefits of implementing 3D printing and AI in healthcare.

Cost-effectiveness analyses reveal variable results depending on application context, production volume, and implementation approach [39]. For high-volume, standardized applications, traditional manufacturing may retain cost advantages, while for highly personalized, complex medical devices, 3D printing offers superior value despite potentially higher per-unit costs [40].

The most consistent and quantifiable cost-effectiveness evidence emerges from operating room (OR) time savings. A 2024 meta-analysis encompassing multiple surgical specialties demonstrated that 3D printing in preoperative planning reduces operation time by 19.85% (95% CI: −22.99, −16.71), intraoperative blood loss by 25.73% (95% CI: −31.07, −20.40), and fluoroscopy usage by 23.80% (95% CI: −38.49, −9.10). These reductions carry significant financial implications given that OR time represents one of healthcare’s most expensive resources, with costs ranging from $36 to $150 per minute depending on facility type and complexity [41].

Healthcare institutions must consider not only direct costs but also opportunity costs and strategic value when evaluating 3D printing and AI investments. institutions offering cutting-edge personalized solutions may gain competitive advantages in increasingly consumer-driven healthcare markets [4]. A comprehensive value assessment framework must therefore consider these broader strategic benefits alongside traditional cost metrics when evaluating technology investments. Incorporating intangible benefits such as improved patient satisfaction and enhanced brand reputation into the value assessment framework is crucial [42].

### 3.2. Operational Implementation and Workflow Integration

Successful integration of 3D printing and AI into healthcare operational workflows requires careful consideration of organizational structure, process redesign, and stakeholder engagement. Implementing these technologies disrupts established clinical and administrative processes, necessitating thoughtful change management strategies.

Process mapping and workflow analysis are essential preparatory steps to identify integration points and potential bottlenecks before technology implementation. Agile project management methodologies can be effective in managing the iterative nature of 3D printing and AI implementation projects [3,43,44].

The operational workflow for 3D printing in healthcare involves several clearly defined stages: acquiring patient data, segmenting and processing medical images, optimizing the design, planning production, printing, post-processing, conducting quality control, and finally, applying the printed device in a clinical setting. To ensure quality and scalability, institutions should establish detailed protocols for each phase, specifying roles, responsibilities, and quality standards. Creating standardized data exchange protocols and APIs is key to connecting 3D printing and AI systems with existing healthcare IT infrastructure [45].

Workforce considerations represent a significant operational challenge, as 3D printing and AI technologies require specialized skills often lacking in traditional healthcare settings. Case studies indicate that hybrid roles combining clinical knowledge with technical skills are particularly valuable in bridging operational gaps [18]. Establishing mentorship programs and communities of practice can facilitate knowledge sharing and skill development within the organization [44].

Studies indicate that AI-driven workflow optimization reduces labor costs associated with 3D printing by 30–40%, significantly improving the technology’s cost-effectiveness profile. These reductions materialize through automated parameter optimization, real-time quality control, predictive maintenance that minimizes downtime, and intelligent material usage that reduces waste [46]. AI-assisted preoperative planning further amplifies these benefits. In percutaneous screw reconstruction for periacetabular metastatic lesions, AI-assisted design with 3D-printed guiding frames reduced mean surgical time from 2.3 h to 1.22 h, with 26 of 36 implanted screws positioned exactly as designed and the remaining ten remaining safely within intra-osseous corridors. This represents a 47% time reduction attributable to AI optimization of the surgical workflow [27].

### 3.3. Governance, Quality Management and Regulatory Considerations

Governance of 3D printing and AI-enhanced medical devices is emerging as a critical determinant of safe, effective, and ethically acceptable clinical translation, spanning regulation, quality systems, data/AI oversight, and institutional responsibilities [6]. 3D-printed and AI-optimized devices generally fall under existing medical device law (e.g., EU MDR, US FDA rules), but point-of-care (PoC) manufacturing blurs the line between “manufacturer” and hospital, creating role ambiguity and new oversight needs [47]. Comparative analyses show that Europe, the USA, and Australia still treat many patient-specific 3D-printed devices as “custom-made” with exemptions, yet these regimes have not fully caught up with additive manufacturing, leaving gaps in pre-market approval, liability, IP, and data protection [48].

Quality assurance represents a paramount concern when implementing 3D printing and AI in healthcare settings, particularly given the personalized nature of produced devices and the potential consequences of failure. Healthcare institutions must establish comprehensive quality management systems (QMS) that address the unique challenges of additive manufacturing technologies. Unlike traditional medical device production with established quality control methods, 3D printing introduces variables related to material properties, printing parameters, post-processing techniques, and design validation that require specialized quality protocols. AI systems, while enhancing quality control through automated inspection and parameter optimization, themselves require validation and ongoing monitoring to ensure reliable performance, creating additional complexity in the quality management framework. Implementing Statistical Process Control (SPC) techniques is crucial for monitoring and controlling the variability in 3D printing processes [24,49].

The regulatory landscape for 3D-printed medical devices and AI-driven healthcare technologies continues to evolve, creating compliance challenges for healthcare organizations. Regulatory bodies worldwide, including the FDA in the United States and the European Medicines Agency, have developed frameworks addressing 3D printing in healthcare. Adopting a “quality by design” (QbD) approach, which emphasizes understanding and controlling critical process parameters, can facilitate regulatory compliance [48,50].

Recent U.S. FDA activity builds on its guidance “Technical Considerations for Additive Manufactured Medical Devices,” which clarifies that 3D-printed products are regulated under existing device pathways and must meet standard quality system, biocompatibility, and sterility requirements, regardless of whether they are printed by industry or in hospitals. For in-house and point-of-care (PoC) 3D printing, the FDA proposes a scenario-based framework that distinguishes low-risk hospital printing, manufacturer-designed devices printed on-site, co-located manufacturers, and hospitals that effectively become full manufacturers, with corresponding expectations for regulatory obligations and quality systems, although this remains discussion rather than binding guidance [51]. Under the EU Medical Device Regulation (MDR), 3D-printed devices are in principle treated like any other device class, but hospital in-house manufacturing is only exempt from full MDR requirements under the strict Article 5(5) conditions for “in-house” devices (non-industrial scale, specific patient needs, documented justification, and an appropriate quality management system), while 3D-printed custom-made devices still require a manufacturer’s declaration, technical documentation, and post-market surveillance, and must comply with general safety and performance requirements even if exempt from CE marking [52].

Documentation and traceability present particular challenges in 3D printing implementations, requiring robust systems to maintain records of design specifications, production parameters, material batches, quality test results, and clinical applications for each produced item. Healthcare management must establish governance structures that clearly define accountability for quality outcomes while creating mechanisms for adverse event reporting and investigation. Forward-thinking organizations are implementing digital quality management systems that integrate with design and production software to automate documentation while ensuring data integrity throughout the product lifecycle. These systems represent a significant operational investment but are increasingly recognized as essential infrastructure for scaling 3D printing applications in healthcare settings. Blockchain technology can enhance traceability and data integrity in 3D printing supply chains [53].

## 4. Personalized Medicine and Patient-Specific Devices

**Figure 3:**
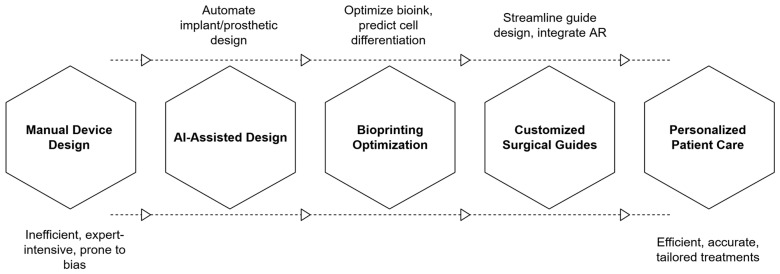
AI-driven translational pipeline from manual medical device design to personalized patient care, highlighting automation, bioprinting optimization, and integration of customized surgical guides.

One of the most impactful applications of 3D printing in healthcare is the creation of patient-specific devices, such as implants, prosthetics, orthoses, and anatomical models, which fall under the umbrella of personalized medicine. AI greatly amplifies the potential of such personalization by automating and refining the design process based on patient data (Figure 3).

### 4.1. AI-Assisted Design of Implants and Prosthetics

Traditionally, designing a custom implant or prosthetic required extensive expert effort to model the patient’s anatomy. The development of AI algorithms that can automatically detect and correct image artifacts in medical images is crucial for ensuring the accuracy of 3D-printed models [54,55,56].

AI is also employed in generative design for prosthetics and orthoses. Machine learning algorithms can optimize the internal lattice structure of a 3D-printed implant to achieve desired strength and weight characteristics or propose novel shapes that distribute stress more evenly [3,27].

AI can further enhance these efforts by analyzing sensor data or user feedback to iteratively improve prosthetic designs (for instance, adjusting finger positions or grip mechanics based on how a patient uses the device). While much of the design of low-cost prosthetics has been manual or open-source, there is growing research on applying AI to biomechanical modeling so that prosthetics can be not only custom-fitted but also functionally optimized for each user’s movement patterns. Integrating haptic feedback into prosthetic design, guided by AI algorithms, can enhance the user’s sense of touch and improve dexterity [57,58].

In AI-assisted implant design, fairness therefore becomes a design constraint across the entire pipeline, rather than a post hoc performance tweak. Mitigation strategies include bias-aware dataset assembly and augmentation; subgroup-stratified training and evaluation; and multi-site or federated learning to reduce site and vendor dependence, complemented by frameworks such as RIDGE that expand evaluation beyond single global metrics. Governance structures must treat segmentation and design models as part of the manufacturing process, requiring routine bias audits, explicit documentation of fairness–accuracy trade-offs, and continuous post-market surveillance to detect performance drifts that may disproportionately affect specific populations [59].

### 4.2. Bioprinting and Regenerative Medicine

An emerging frontier of personalized medicine is 3D bioprinting (printing with cells and bio-materials to create tissues or organs for a specific patient). AI is being applied here to address the complexity of biological systems. Recent reviews highlight that integrating AI into bioprinting can improve the personalization of design and scaling up of production for regenerative products. For instance, machine learning models have been used to optimize bioink formulations (the mixtures of cells and biomaterials) by predicting how changes at the molecular or microstructural level will affect the printability and viability of the tissue. The use of AI to predict the differentiation pathways of stem cells within bioprinted constructs is crucial for creating functional tissues [32,60].

When designing bioprinted constructs (like a tissue scaffold for a specific patient’s organ), AI can aid in multi-scale modeling—first interpreting medical images to capture the macro-scale shape needed, and then helping design the micro-architecture (e.g., vascular channels, pore networks) that will promote cell growth. AI will be crucial in this domain to handle the tremendous complexity (billions of cells in structured arrangements) and to monitor prints in real time, possibly with reinforcement learning controllers adjusting parameters to keep cells alive and correctly placed. The synergy of AI and bioprinting thus holds promise to eventually fabricate transplantable tissues or organoids tailored to individual patients, a highly personalized form of therapy. Developing AI algorithms that can predict the long-term viability and functionality of bioprinted tissues after implantation is a critical research priority [61].

### 4.3. Customized Surgical Guides and Tools

Another personalized application is the production of patient-specific surgical guides, jigs, and tools that help surgeons perform operations more precisely. AI can streamline the design of such guides by automatically identifying the optimal placement and geometry based on the patient’s anatomy and the surgical plan. While the design in those cases may have been manual, we can envision AI tools that take a planned surgical trajectory and generate a guide model ready for 3D printing, needing only minor surgeon approval. This kind of automation would integrate patient-specific tool fabrication seamlessly into the surgical workflow, enhancing precision and reducing costs (since shorter surgeries not only save money but also reduce anesthesia and complication risks). Integrating augmented reality (AR) with 3D-printed surgical guides can provide surgeons with real-time visual feedback during procedures, further enhancing precision [29,40].

## 5. Customized Surgical Guides and Tools

### 5.1. AI-Driven Optimization of 3D Printing Resources

Effective healthcare management hinges on streamlined supply chains and logistics, ensuring that medical supplies and devices are available when and where they are needed. AI-driven analytics can optimize the utilization of 3D printing resources, reducing inventory costs, minimizing waste, and improving responsiveness to urgent needs. Predictive models can forecast demand for specific medical devices or supplies based on historical data, seasonal trends, and real-time patient needs [62]. These forecasts can guide production planning, ensuring that 3D printers are utilized efficiently and that inventory levels are aligned with demand. The integration of real-time data from electronic health records (EHRs) and inventory management systems is crucial for accurate demand forecasting [63].

AI can also optimize the allocation of 3D printers within a healthcare network, ensuring that resources are deployed strategically to meet the needs of different departments or facilities. Dynamic routing algorithms can optimize the delivery of 3D-printed medical devices and supplies within a healthcare network, reducing transportation costs and improving responsiveness to urgent needs. The use of drone delivery systems, guided by AI algorithms, can further accelerate the delivery of critical medical supplies in remote or difficult-to-reach areas.

### 5.2. On-Demand Production and Point-of-Care Manufacturing

One of the key advantages of 3D printing is its ability to enable on-demand production of medical devices and supplies at the point of care. This can be particularly valuable in situations where there are supply chain disruptions, limited inventory, or urgent patient needs. AI can facilitate on-demand production by automating the design and manufacturing processes, ensuring that medical devices and supplies can be produced quickly and efficiently. The integration of 3D printing with mobile health (mHealth) technologies can enable remote monitoring of patient conditions and on-demand production of personalized medical devices at home [1,64].

Point-of-care manufacturing requires careful consideration of regulatory and quality control issues. Healthcare institutions must establish comprehensive quality management systems to ensure that 3D-printed medical devices and supplies meet the required standards for safety and effectiveness. AI can enhance quality control by automating inspection processes, detecting defects, and optimizing printing parameters. The use of blockchain technology can enhance traceability and data integrity in the 3D printing supply chain, ensuring that all medical devices and supplies can be tracked from design to delivery [50].

### 5.3. AI-Enabled Inventory Management and Waste Reduction

Effective inventory management is essential for healthcare facilities, ensuring that medical supplies and devices are available when needed while also minimizing waste and storage costs. AI-powered analytics can optimize inventory by predicting demand, identifying slow-moving items, and automating reordering processes. The use of RFID (radio-frequency identification) tags and sensor networks provides real-time visibility into inventory quantities and locations, enabling more efficient management of medical supplies and devices. Integrating AI with robotic systems can further automate inventory tasks, such as restocking shelves, retrieving items, and performing cycle counts [65].

AI can also play a key role in reducing waste in healthcare supply chains. By predicting demand and optimizing inventory levels, healthcare facilities can minimize the risk of spoilage or expiration of medical supplies and devices. AI algorithms can analyze data on product usage and expiration dates to identify items that are at risk of becoming obsolete, allowing healthcare facilities to take proactive measures to prevent waste. The use of 3D printing can further reduce waste by enabling on-demand production of medical devices and supplies, eliminating the need to stockpile inventory. The development of closed-loop recycling systems for 3D-printed materials can further enhance sustainability and reduce waste [66].

## 6. Global Perspectives and Regional Variations

### 6.1. Adoption Patterns Across Healthcare Systems

The integration of 3D printing and AI technologies in healthcare exhibits significant variation across global regions, reflecting differences in healthcare system structures, funding mechanisms, and technological infrastructure. High-resource settings, particularly in North America, Western Europe, and parts of East Asia, have generally led early adoption, benefiting from established research institutions, technological expertise, and capital resources. In these regions, academic medical centers typically function as innovation hubs, developing applications subsequently diffused to community hospitals and private healthcare providers. The development of international standards for 3D-printed medical devices can facilitate global adoption and interoperability [45,67].

Middle-income countries display a varied landscape. While centers of excellence have achieved sophisticated implementations, much of the broader healthcare system continues to face resource constraints that limit widespread adoption. For example, countries such as Malaysia, Thailand, and regions of China have established specialized centers with advanced 3D printing capabilities, often focusing on high-value applications that address healthcare challenges specific to their populations. Additionally, using open-source software and hardware can further lower the cost of implementing 3D printing and AI technologies in resource-constrained settings [68].

Low-resource settings face substantial challenges in technology adoption, including limited capital for equipment acquisition, insufficient technical expertise, and competing priorities for basic healthcare infrastructure. Nevertheless, innovative implementations have emerged, often focusing on specific high-impact applications such as prosthetics production in regions with high amputation rates due to conflict or disease. Organizations in these settings frequently adapt consumer-grade equipment and open-source software to reduce costs while developing simplified workflows suitable for environments with limited technical support [69].

International collaborations, non-governmental organizations, and academic partnerships play crucial roles in facilitating technology transfer and capacity building. These resource-constrained implementations often demonstrate remarkable ingenuity in adapting technologies to local contexts, sometimes generating innovations subsequently adopted in higher-resource settings. The development of culturally appropriate 3D-printed medical devices is crucial for ensuring patient acceptance and adherence in diverse populations.

### 6.2. Sustainable Healthcare Management Approaches

Sustainability considerations are increasingly influencing the implementation of 3D printing and AI technologies in healthcare systems worldwide. Environmental sustainability represents a growing concern, as traditional healthcare manufacturing generates substantial waste through mass production of standard devices, many of which remain unused. Additive manufacturing offers potential advantages through on-demand production that eliminates overstock waste while reducing transportation-related carbon emissions associated with global supply chains. However, the environmental impacts of 3D printing processes themselves require careful consideration, including energy consumption, material waste, and end-of-life considerations for produced devices. Healthcare organizations implementing these technologies are increasingly adopting life-cycle assessment methodologies to evaluate environmental impacts comprehensively rather than focusing solely on production-phase considerations. The use of biodegradable and recyclable materials in 3D printing can further enhance environmental sustainability [1,70].

Economic sustainability necessitates business models that balance technology investments with demonstrable value creation, ensuring long-term viability beyond initial implementation. For high-volume, standardized devices and dosage forms, traditional manufacturing methods such as injection molding or conventional pharmaceutical processing still deliver lower per-unit costs, higher throughput, and very mature quality systems, making them economically and operationally preferable. In addition, 3D printing remains constrained by a narrower palette of biocompatible, sterilizable materials, longer production times, and a substantial quality assurance burden, since seemingly small changes in design or process parameters can alter mechanical and biological performance and demand rigorous re-validation. These limitations mean that for commodity products with established global supply chains and proven long-term reliability, switching to additive manufacturing may add complexity without corresponding clinical or economic benefit [66].

Social sustainability encompasses considerations of access equity, workforce development, and community engagement. As 3D printing and AI technologies enhance treatment options, healthcare systems must ensure these benefits extend across socioeconomic boundaries rather than exacerbating existing healthcare disparities. Implementation strategies emphasizing knowledge transfer and capacity building contribute to social sustainability by developing local expertise rather than creating dependence on external specialists. The integration of 3D printing and AI technologies with telehealth platforms can improve access to healthcare services in remote and underserved areas [1,4].

### 6.3. Cross-Cultural Considerations in Technology Implementation

Cultural factors significantly influence the acceptance, utilization, and effectiveness of 3D printing and AI technologies across different healthcare contexts. Attitudes toward technology, perceptions of personalized medicine, and comfort with AI-assisted decision-making vary substantially across cultural settings, necessitating culturally sensitive implementation approaches. In some settings, advanced technologies carry significant prestige, potentially accelerating patient acceptance while creating unrealistic expectations about outcomes. Conversely, in contexts with strong traditions of artisanal craftsmanship, the machine-made nature of 3D printed devices may initially generate skepticism regarding quality and appropriateness, requiring educational interventions that emphasize the human expertise underlying design processes. The development of culturally appropriate communication materials is crucial for ensuring patient understanding and engagement [12,71].

The culture among healthcare professionals significantly influences technology adoption, with different clinical specialties and professional groups responding in varied ways to innovation. Surgical disciplines have typically adopted 3D printing technologies more quickly than non-procedural fields, largely due to the direct benefits for surgical planning and procedures. When organizations overlook these cultural aspects, they often encounter implementation failures despite having sufficient technical and financial resources. Forming interdisciplinary teams—including clinicians, engineers, and ethicists—can help ensure culturally sensitive and successful technology adoption [18].

Language and communication factors present another important cross-cultural consideration, particularly for AI systems trained primarily on English-language medical literature and terminology. Natural language processing capabilities may perform poorly when applied in non-English healthcare settings or with regional linguistic variations, potentially limiting the effectiveness of AI-assisted design systems. Successful global implementations adapt not only technical aspects but also communication approaches to align with local preferences and practices. These adaptations require genuine cultural competence rather than superficial localization, highlighting the importance of diverse development teams and extensive stakeholder engagement across implementation phases. The use of machine translation technologies can improve communication and collaboration in multilingual healthcare environments [69,72].

## 7. Ethical and Legal Frameworks

### 7.1. Patient Privacy and Data Security

**Figure 4:**
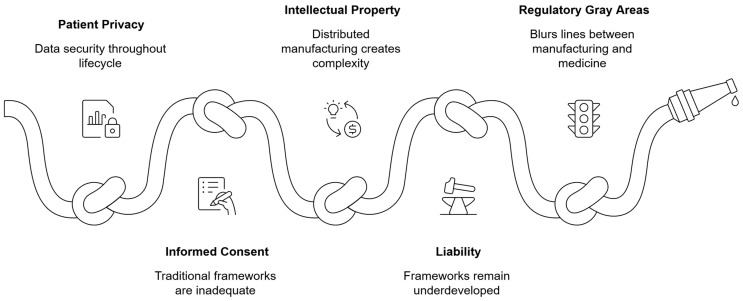
Conceptual mapping of ethical and legal constraints affecting the adoption of AI-enabled 3D printing technologies in healthcare.

The integration of 3D printing and AI in healthcare creates novel challenges for patient privacy and data security throughout the data lifecycle. Patient-specific medical devices require detailed anatomical data derived from medical imaging, creating potential vulnerability points for protected health information (Figure 4). The digital workflow encompasses numerous data transfer points, from imaging departments to design software to production facilities, each presenting privacy risks if not properly secured. Furthermore, the physical outputs themselves may contain identifiable patient information in their very structure, requiring consideration of privacy implications even after production is complete. Healthcare organizations must implement comprehensive data governance frameworks that address these unique challenges while complying with regulations such as HIPAA in the United States and GDPR in Europe. The implementation of data encryption and access control mechanisms is crucial for protecting patient privacy [48].

AI systems present additional privacy considerations, particularly when using patient data for algorithm training and validation. The extensive datasets required for effective AI training create tension between data utilization for system improvement and patient privacy protection. Techniques such as federated learning, differential privacy, and synthetic data generation are emerging as potential solutions that balance these competing priorities, though each presents implementation challenges and potential limitations. Organizations must establish clear policies regarding data ownership, consent requirements, and appropriate use limitations, particularly when collaborating with external technology providers or research institutions. Transparency with patients regarding how their data contributes to technology development represents both an ethical obligation and a practical necessity for maintaining trust. The use of de-identified data and anonymization techniques can reduce the risk of patient identification [72].

Security vulnerabilities in connected manufacturing systems require particular attention as healthcare organizations increasingly implement networked 3D printing capabilities. These systems may be susceptible to various attack vectors, including design file manipulation, parameter tampering, or unauthorized access to protected health information. Security frameworks must address physical security of production facilities, network security for connected equipment, authentication mechanisms for design approval workflows, and integrity verification for production files and parameters. The implementation of intrusion detection and prevention systems is crucial for protecting 3D printing and AI systems from cyberattacks [53].

### 7.2. Informed Consent and Patient Autonomy

The use of personalized 3D printed medical devices and AI-guided treatment planning raises important questions regarding informed consent processes and patient autonomy. Traditional informed consent frameworks may inadequately address the unique aspects of these technologies, including algorithmic decision support, novel materials, and limited long-term outcomes data. Visual aids such as 3D printed models can actually improve patient understanding of proposed procedures, potentially enhancing the quality of consent, though care must be taken to present realistic expectations regarding outcomes and limitations. The use of decision support tools can help patients make informed choices about their treatment options [48,73].

Patient involvement in design decisions represents another dimension of autonomy that requires careful consideration. The customization capabilities of 3D printing technology potentially allow greater patient input regarding device characteristics, functional priorities, and aesthetic considerations. These approaches recognize patient expertise regarding their own experiences and priorities while respecting clinical expertise regarding technical requirements. The development of user-friendly design interfaces can empower patients to participate in the design process [5,50].

The “right to explainability” presents particular challenges when AI systems influence treatment decisions or device designs. Patients may reasonably expect understandable explanations of how algorithms contributed to their care plans, yet many current AI systems operate as “black boxes” with limited explainability. Developing appropriate frameworks for AI explainability in patient care contexts represents an important area for collaborative work between clinicians, ethicists, and technology developers, with potential regulatory implications as healthcare AI applications continue to expand. The use of visualization techniques can help patients understand the reasoning behind AI-driven recommendations [48].

### 7.3. Intellectual Property and Liability Considerations

The distributed manufacturing capabilities enabled by 3D printing technologies create complex intellectual property questions for healthcare organizations. When hospitals produce medical devices on-site using designs from various sources, questions arise regarding design ownership, licensing requirements, and appropriate compensation for intellectual property holders. Various models have emerged, including subscription-based access to design libraries, per-use licensing fees, and open-source approaches for specific applications. Healthcare organizations must establish clear policies regarding design sourcing, modification rights, and attribution requirements to ensure compliance with intellectual property laws while maintaining necessary flexibility for clinical applications. Additionally, tensions may arise between proprietary approaches that protect commercial interests and open-source philosophies that potentially accelerate innovation and improve access. The use of blockchain technology can facilitate intellectual property management and licensing in 3D printing [50,74].

Liability frameworks for patient-specific medical devices produced using 3D printing and AI technologies remain underdeveloped in many jurisdictions, creating uncertainty for healthcare providers. When adverse events occur involving personalized devices, questions arise regarding liability distribution among treating physicians, design engineers, manufacturing technicians, software developers, and the healthcare institution itself. Healthcare organizations implementing these technologies must develop risk management strategies addressing this uncertainty while ensuring appropriate insurance coverage for novel liability scenarios. Detailed documentation of design decisions, production parameters, quality verification procedures, and clinical reasoning becomes particularly important in this context. The implementation of failure mode and effects analysis (FMEA) can help identify potential risks and vulnerabilities in 3D printing processes [53,75].

Regulatory classifications significantly influence liability considerations, yet many 3D printing applications exist in regulatory gray areas. The FDA’s distinction between commercial manufacturing and “practice of medicine” activities becomes blurred when healthcare institutions engage in point-of-care manufacturing of patient-specific devices. Regulatory frameworks worldwide are evolving to address these scenarios, though significant variations exist across jurisdictions, creating compliance challenges for multinational healthcare organizations. Proactive engagement with regulatory authorities through pre-submission consultations and participation in standard-setting initiatives represents a prudent approach for healthcare institutions pioneering these technologies. The development of standardized testing and validation methods for 3D-printed medical devices can reduce the risk of product liability claims [8,75,76].

## 8. Challenges and Future Directions

### 8.1. Implementation Barriers and Adoption Strategies

Although 3D printing and AI technologies offer clear advantages, several significant barriers hinder their widespread adoption in healthcare. Financial challenges are a major obstacle, especially for smaller institutions with limited capital budgets and uncertainty about the timeline for return on investment. The substantial upfront costs for equipment, software licenses, and specialized personnel can seem prohibitive without well-defined reimbursement options. Technical complexity also poses a considerable challenge, as many healthcare organizations lack in-house expertise in advanced manufacturing and computational design. Additionally, cultural resistance often arises among clinicians who are accustomed to traditional methods, particularly when new technologies disrupt established workflows without providing immediately obvious benefits. Overcoming these varied barriers requires comprehensive implementation strategies that are carefully tailored to each organization’s specific circumstances [77].

Effective adoption strategies begin with strategic alignment, ensuring 3D printing and AI initiatives support institutional priorities rather than functioning as isolated technological experiments. Organizations that successfully implement these technologies typically start with high-impact, focused applications that demonstrate clear value, gradually expanding scope as expertise and confidence develop. Establishing mentorship programs and communities of practice can facilitate knowledge sharing and skill development within the organization [18].

Education and training emerge as critical enablers of successful adoption, addressing both technical skill development and conceptual understanding of technology applications. Comprehensive educational programs must target multiple stakeholder groups, including clinical staff, administrators, technical personnel, and patients. Research indicates that hands-on workshops, simulation exercises, and peer mentoring are particularly effective in building competence and confidence among healthcare professionals. Developing online learning platforms and virtual reality (VR) simulations can enhance accessibility and engagement in education and training programs [36,78].

### 8.2. Emerging Trends and Future Applications

The confluence of 3D printing and artificial intelligence continues to generate novel applications with significant implications for healthcare management. Bioprinting represents one of the most promising frontier technologies, moving beyond structural applications toward functional tissue engineering. The development of personalized drug delivery systems, using 3D printing and AI, can optimize treatment outcomes and reduce side effects [79,80].

The combination of 3D printing, Internet of Things (IoT) technologies, and AI-driven analytics is opening new opportunities for smart medical devices equipped with monitoring and adaptive features. By embedding sensors into 3D-printed medical devices, healthcare providers can collect real-time data about device performance, patient physiology, and treatment progress. AI algorithms then analyze this data to detect patterns, predict potential complications, and recommend interventions before a patient’s condition worsens. For instance, smart orthopedic implants can track load distribution, identify early signs of loosening, and help guide adjustments to rehabilitation protocols. These intelligent medical devices mark a transition from passive to active therapy, offering important benefits for chronic disease management and remote care. Additionally, blockchain technology can be used to protect data security and privacy in IoT-connected, 3D-printed medical devices [81].

Space medicine represents an emerging application domain with unique challenges and opportunities for 3D printing and AI technologies. On-demand pharmaceutical and device fabrication concepts developed for long-duration missions (e.g., small-footprint, modular, and quality-controlled production of medicines and components) offer a blueprint for regionalized or point-of-care manufacturing networks that can buffer fragile supply chains in remote areas or disaster zones, provided that simplified yet stringent quality and maintenance frameworks are put in place [82].

### 8.3. Research Gaps and Future Directions

Despite significant progress, substantial research gaps remain in understanding the full potential and optimal implementation approaches for 3D printing and AI in healthcare management. Long-term clinical outcomes data for patients treated with 3D printed devices remains limited, particularly comparative effectiveness studies against traditional approaches. Economic analyses frequently focus on direct production costs while overlooking broader system impacts such as reduced complication rates, shortened hospital stays, and improved functional outcomes. Implementation science research examining organizational factors influencing successful adoption is particularly underdeveloped, leaving healthcare leaders without evidence-based guidance for change management strategies. Additionally, standardized methods for quality assurance and validation specific to healthcare 3D printing applications require further development to support regulatory compliance and risk management. The development of digital twins for 3D-printed medical devices can enable virtual testing and optimization, reducing the need for physical prototypes [2,54,79].

Material science represents another critical research frontier, as current printing materials often present limitations for specific healthcare applications. Biocompatible materials with appropriate mechanical properties, degradation profiles, and drug-eluting capabilities require continued development for applications ranging from implantable devices to tissue engineering scaffolds. The use of nanotechnology can enhance the properties of 3D-printed materials, improving their strength, biocompatibility, and functionality [2,83].

Artificial intelligence applications specific to healthcare manufacturing require further research, particularly regarding explainable AI systems that enable verification of design decisions—critical for regulatory approval and clinical confidence. Current deep learning approaches often function as “black boxes,” making their decision processes opaque to human reviewers. Developing AI systems that provide transparent reasoning for design modifications would accelerate clinical adoption while facilitating regulatory review. The development of federated learning algorithms can enable AI training on distributed datasets without compromising patient privacy [48,50].

### 8.4. Limitations

For 3D printing in healthcare, the current evidence base is promising but heterogeneous, with substantial variation in study design, sample size, endpoints, and quality. Systematic reviews of clinical efficacy highlight that while 3D-printed guides, models, and implants often outperform conventional comparators in specific areas such as orthopedic and maxillofacial surgery, the effectiveness of 3D-printed solutions remains undetermined in many other specialties, and long-term, high-quality outcome data are still scarce. Quality assurance reviews similarly show wide variation in how segmentation error, digital editing error, and printing error are measured and reported, underlining the need for standardized QA frameworks and stronger methodological rigor before findings can be generalized across settings. Regulatory and liability uncertainty is particularly salient for point-of-care manufacturing, where hospitals increasingly act as “manufacturers” but operate under evolving MDR/FDA interpretations, creating ambiguity around responsibility for device performance, software validation, and incident reporting [18].

In the short term, priorities include robust workflow integration into existing clinical pathways, development of decision-support and indication-selection tools for when 3D printing truly adds clinical value, and implementation of context-based quality assurance standards that are practical for point-of-care settings.

Medium-term developments focus on scalable deployment models, standardized governance and regulatory-compliance frameworks, and more rigorous economic evaluations that explicitly distinguish clinical value—such as gains in precision, personalization, and outcome improvement—from healthcare management value, including more efficient resource allocation, operational resilience, and clearer accountability structures.

Over the longer term, directions such as the maturation of bioprinting for tissue and organ constructs, increasingly autonomous AI-driven design pipelines, and deeper integration of distributed manufacturing into system-wide resilience strategies (e.g., crisis response, supply chain redesign) will require not only technical breakthroughs but also sophisticated management and policy frameworks that reconcile innovation with safety, equity, and financial sustainability [68].

## 9. Conclusions

The integration of 3D printing and artificial intelligence has the potential to transform healthcare management by enabling greater personalization, operational efficiency, and resource optimization. This review highlights how these technologies can support decision-making, cost containment, and care delivery across diverse healthcare contexts, while also revealing significant technical, organizational, ethical, and regulatory challenges that influence successful adoption.

Effective implementation requires more than technological capability. Healthcare organizations must adopt structured strategies that address workforce development, change management, governance, and performance monitoring. Phased deployment, interdisciplinary collaboration, and the use of measurable indicators are essential to align innovation with clinical and managerial value.

Future research should prioritize robust evidence on long-term clinical and economic outcomes, scalable implementation models, and governance frameworks suitable for both high- and resource-constrained settings. Addressing these gaps will be critical to ensuring that AI-enabled 3D printing contributes sustainably and equitably to healthcare management.

## Figures and Tables

**Table 1:** Overview of artificial intelligence techniques applied to 3D printing in healthcare and their associated management impacts.

AI Technique	3D Printing Application	Healthcare Management Impact
Machine learning (supervised/unsupervised)	Patient-specific implants and prosthetics	Improved decision support for device selection; reduced revision rates; better resource utilization
Deep learning (CNNs)	Medical image segmentation for surgical models	Faster workflow integration; reduced preoperative planning time; increased operational efficiency
Generative design algorithms	Implant and prosthetic design optimization	Cost reduction through material optimization; shorter design cycles; enhanced value-based care
Reinforcement learning	Process control and printer parameter optimization	Improved quality assurance; reduced waste and rework; increased operational resilience
Computer vision	Real-time print monitoring and defect detection	Enhanced governance and accountability; standardized quality control processes
Predictive analytics	Demand forecasting for point-of-care manufacturing	Improved inventory management; reduced stockpiling; supply chain resilience
Natural language processing (NLP)	Identification of cases suitable for 3D printing from clinical records	Decision support for managers; improved workflow prioritization; automation of service requests
AI-assisted simulation and modeling	Surgical planning and outcome prediction	Risk reduction; improved clinical and managerial decision-making; reduced operating time
Machine learning–driven bioink optimization	Bioprinting and regenerative medicine	Long-term capacity planning; R&D efficiency; strategic investment support

## Data Availability

No new data were created.

## Abbreviations

The following abbreviations are used in this manuscript:

3D	Three-Dimensional
4D	Four-Dimensional
AI	Artificial Intelligence
AM	Additive Manufacturing
API	Application Programming Interface
AR	Augmented Reality
CNN	Convolutional Neural Network
CT	Computed Tomography
DFU	Diabetic Foot Ulcer
DLP	Digital Light Processing
EHR	Electronic Health Record
FDA	Food and Drug Administration
FDM	Fused Deposition Modeling
FMEA	Failure Mode and Effects Analysis
GDPR	General Data Protection Regulation
HIPAA	Health Insurance Portability and Accountability Act
IoT	Internet of Things
KPI	Key Performance Indicator
ML	Machine Learning
MRI	Magnetic Resonance Imaging
mHealth	Mobile Health
NLP	Natural Language Processing
PPE	Personal Protective Equipment
QbD	Quality by Design
QMS	Quality Management System
RFID	Radio-Frequency Identification
RL	Reinforcement Learning
RWE	Real-World Evidence
SLA	Stereolithography
SLS	Selective Laser Sintering
SPC	Statistical Process Control
TCO	Total Cost of Ownership
U-Net	U-Net Neural Network Architecture
VR	Virtual Reality

## References

[1] Urbaite G. (2024). 3D Printing and Additive Manufacturing: Revolutionizing the Production Process. Luminis Appl. Sci. Eng..

[2] Schwam Z.G., Chang M.T., Barnes M.A., Paskhover B. (2016). Applications of 3-Dimensional Printing in Facial Plastic Surgery. J. Oral Maxillofac. Surg..

[3] Wixted C.M., Peterson J.R., Kadakia R.J., Adams S.B. (2021). Three-Dimensional Printing in Orthopaedic Surgery: Current Applications and Future Developments. J. Am. Acad. Orthop. Surg. Glob. Res. Rev..

[4] Martelli N., Serrano C., van den Brink H., Pineau J., Prognon P., Borget I., El Batti S. (2016). Advantages and Disadvantages of 3-Dimensional Printing in Surgery: A Systematic Review. Surgery.

[5] Satapathy S.R., Sahoo R.N., Nandi S., Satapathy B., Panigrahi L., Mallick S. (2021). 3D Printing in Managing Supply Disruptions Related to COVID-19 Pandemic: Food and Drug Administration’s Current Thinking on Regulation. Minerva Biotechnol. Biomol. Res..

[6] Rojek I., Mikołajewski D., Dostatni E., Kopowski J. (2023). Specificity of 3D Printing and AI-Based Optimization of Medical Devices Using the Example of a Group of Exoskeletons. Appl. Sci..

[7] Rebahi Y., Gharra M., Rizzi L., Zournatzis I. (2023). Combining Computer Vision, Artificial Intelligence and 3D Printing in Wheelchair Design Customization: The Kyklos 4.0 Approach. Artif. Intell. Appl..

[8] Rahman Z., Barakh Ali S.F., Ozkan T., Charoo N.A., Reddy I.K., Khan M.A. (2018). Additive Manufacturing with 3D Printing: Progress from Bench to Bedside. AAPS J..

[9] Banerjee A., Haridas H., Sengupta A., Jabalia N. (2021). Artificial Intelligence in 3D Printing: A Revolution in Health Care.

[10] Sokmen S., Cakmak S., Oksuz I. (2024). 3D Printing of an Artificial Intelligence-Generated Patient-Specific Coronary Artery Segmentation in a Support Bath. Biomed. Mater..

[11] Ballard D.H., Mills P., Duszak R., Weisman J.A., Rybicki F.J., Woodard P.K. (2020). Medical 3D Printing Cost-Savings in Orthopedic and Maxillofacial Surgery: Cost Analysis of Operating Room Time Saved with 3D Printed Anatomic Models and Surgical Guides. Acad. Radiol..

[12] Thacharodi A., Singh P., Meenatchi R., Tawfeeq Ahmed Z.H., Kumar R.R.S., V N., Kavish S., Maqbool M., Hassan S. (2024). Revolutionizing Healthcare and Medicine: The Impact of Modern Technologies for a Healthier Future—A Comprehensive Review. Health Care Sci..

[13] Carvalho V., Gonçalves I., Lage T., Rodrigues R.O., Minas G., Teixeira S.F.C.F., Moita A.S., Hori T., Kaji H., Lima R.A. (2021). 3D Printing Techniques and Their Applications to Organ-on-a-Chip Platforms: A Systematic Review. Sensors.

[14] Anagnostopoulos S., Gallos P., Zoulias E., Fotos N., Mantas J., Mantas J., Gallos P., Zoulias E., Hasman A., Househ M.S., Diomidous M., Liaskos J., Charalampidou M. (2022). 3D Digital Printing in Healthcare: Technologies, Applications and Health Issues. Studies in Health Technology and Informatics.

[15] Ravi P., Chepelev L.L., Stichweh G.V., Jones B.S., Rybicki F.J. (2022). Medical 3D Printing Dimensional Accuracy for Multi-Pathological Anatomical Models 3D Printed Using Material Extrusion. J. Digit. Imaging.

[16] Jeong Y.G., Yoo J.J., Lee S.J., Kim M.S. (2024). 3D Digital Light Process Bioprinting: Cutting-Edge Platforms for Resolution of Organ Fabrication. Mater. Today Bio.

[17] Abolhassani A., Jones T., Bhatt A.N., McClain J., Ahmed A., Davis J., Bethel M. (2025). A Decade in Print: The Evolving Academic Benchmark of Cardiology Fellowship Applications. Cureus.

[18] Ikhsan R.Z., Rahayu S., Arribathi A.H., Azizah N. (2024). Integrating Artificial Intelligence with 3D Printing Technology in Healthcare: Sustainable Solutions for Clinical Training Optimization. ADI J. Recent Innov. (AJRI).

[19] Khandare M.S.N., Kadu M.A., Kaware M.P., Kaldate M.R., Solav M.A. (2024). Integration into 3D Printing for Image Processing Using AI ML. Int. J. Adv. Res. Sci. Commun. Technol..

[20] Agarwal A., Kumar R., Gupta M. (2022). Review on Deep Learning Based Medical Image Processing. Proceedings of the 2022 IEEE International Conference on Current Development in Engineering and Technology (CCET), Bhopal, India, 23–24 December 2022.

[21] Teng Z., Li L., Xin Z., Xiang D., Huang J., Zhou H., Shi F., Zhu W., Cai J., Peng T. (2024). A Literature Review of Artificial Intelligence (AI) for Medical Image Segmentation: From AI and Explainable AI to Trustworthy AI. Quant. Imaging Med. Surg..

[22] Sha A., R A.K.E., Menon D.S., T A. (2023). Enhancing Segmentation Efficiency: A 2D U-Net Approach with 3D-to-2D Conversion for Medical Image Analysis. Proceedings of the 2023 7th International Conference on Electronics, Communication and Aerospace Technology (ICECA), Coimbatore, India, 22–24 November 2023.

[23] Paraskevoudis K., Karayannis P., Koumoulos E.P. (2020). Real-Time 3D Printing Remote Defect Detection (Stringing) with Computer Vision and Artificial Intelligence. Processes.

[24] Batra R., Mittal G., Saha A. (2023). An Organized Review of Machine Learning (ML) Perspectives in Manufacturing and Quality Control Processes. Proceedings of the 2023 International Conference on Power Energy, Environment & Intelligent Control (PEEIC), Greater Noida, India, 19–23 December 2023.

[25] Akmal J.S., Salmi M., Hemming B., Teir L., Suomalainen A., Kortesniemi M., Partanen J., Lassila A. (2020). Cumulative Inaccuracies in Implementation of Additive Manufacturing Through Medical Imaging, 3D Thresholding, and 3D Modeling: A Case Study for an End-Use Implant. Appl. Sci..

[26] Moroni S., Casettari L., Lamprou D.A. (2022). 3D and 4D Printing in the Fight against Breast Cancer. Biosensors.

[27] Wang J., Zhao Z., Liang H., Zhang R., Liu X., Zhang J., Singh S., Guo W., Yan T., Hoang B.H. (2024). Artificial Intelligence Assisted Preoperative Planning and 3D-Printing Guiding Frame for Percutaneous Screw Reconstruction in Periacetabular Metastatic Cancer Patients. Front. Bioeng. Biotechnol..

[28] Gómez V.J., Martín-González A., Zafra-Vallejo V., Zubillaga-Rodríguez I., Fernández-García A., Sánchez-Aniceto G. (2023). In-House Virtual Surgery Planning and 3D Printing for Head and Neck Surgery with Free Software: Our Workflow. Craniomaxillofacial Trauma Reconstr..

[29] Suleman A., Kondiah P.P.D., Mabrouk M., Choonara Y.E. (2021). The Application of 3D-Printing and Nanotechnology for the Targeted Treatment of Osteosarcoma. Front. Mater..

[30] Zhang Q., Li Z., Chen Z., Peng Y., Jin Z., Qin L. (2023). Prediction of Knee Biomechanics with Different Tibial Component Malrotations after Total Knee Arthroplasty: Conventional Machine Learning vs. Deep Learning. Front. Bioeng. Biotechnol..

[31] Li X., Ai X., Wang B., Luo M., Miyamoto A., Kuchay M.S., Feng D., Zhang C. (2024). Application of 3D Printing in the Treatment of Diabetic Foot Ulcers: Current Status and New Insights. Front. Bioeng. Biotechnol..

[32] Guptha P.M., Kanoujia J., Kishore A., Raina N., Wahi A., Gupta P.K., Gupta M. (2024). A Comprehensive Review of the Application of 3D-Bioprinting in Chronic Wound Management. Expert Opin. Drug Deliv..

[33] Abuhamad A.Y., Masri S., Fadilah N.I.M., Alamassi M.N., Maarof M., Fauzi M.B. (2024). Application of 3D-Printed Bioinks in Chronic Wound Healing: A Scoping Review. Polymers.

[34] Carey H. (2024). How Artificial Intelligence Is Shaping the Development and Design of Medical Implants. J. Med. Implant..

[35] Dong C., Petrovic M., Davies I.J. (2024). Applications of 3D printing in medicine: A review. Ann. 3D Print. Med..

[36] Meyer-Szary J., Luis M.S., Mikulski S., Patel A., Schulz F., Tretiakow D., Fercho J., Jaguszewska K., Frankiewicz M., Pawłowska E. (2022). The Role of 3D Printing in Planning Complex Medical Procedures and Training of Medical Professionals-Cross-Sectional Multispecialty Review. Int. J. Environ. Res. Public Health.

[37] Baig M.A., Norah A., Haifa A., Nouf A., Baig S.M. (2023). Implementation of 3D Printing in Various Healthcare Settings: A Scoping Review. Stud. Health Technol. Inform..

[38] Paul G.M., Rezaienia A., Wen P., Condoor S., Parkar N., King W., Korakianitis T. (2018). Medical Applications for 3D Printing: Recent Developments. Mo. Med..

[39] Alzoubi L., Aljabali A.A.A., Tambuwala M.M. (2023). Empowering Precision Medicine: The Impact of 3D Printing on Personalized Therapeutic. AAPS PharmSciTech.

[40] Urlings J., de Jong G., Maal T., Henssen D. (2023). Views on Augmented Reality, Virtual Reality, and 3D Printing in Modern Medicine and Education: A Qualitative Exploration of Expert Opinion. J. Digit. Imaging.

[41] Choonara Y.E., du Toit L.C., Kumar P., Kondiah P.P.D., Pillay V. (2016). 3D-Printing and the Effect on Medical Costs: A New Era?. Expert Rev. Pharmacoeconomics Outcomes Res..

[42] Khanna N.N., Maindarkar M.A., Viswanathan V., Fernandes J.F.E., Paul S., Bhagawati M., Ahluwalia P., Ruzsa Z., Sharma A., Kolluri R. (2022). Economics of Artificial Intelligence in Healthcare: Diagnosis vs. Treatment. Healthcare.

[43] Ventola C.L. (2014). Medical Applications for 3D Printing: Current and Projected Uses. Pharm. Ther..

[44] Ramola M., Yadav V., Jain R. (2019). On the Adoption of Additive Manufacturing in Healthcare: A Literature Review. J. Manuf. Technol. Manag..

[45] Shim K.W. (2023). Medical Applications of 3D Printing and Standardization Issues. Brain Tumor Res. Treat..

[46] Pugliese R., Regondi S. (2022). Artificial Intelligence-Empowered 3D and 4D Printing Technologies toward Smarter Biomedical Materials and Approaches. Polymers.

[47] Beitler B.G., Abraham P.F., Glennon A.R., Tommasini S.M., Lattanza L.L., Morris J.M., Wiznia D.H. (2022). Interpretation of Regulatory Factors for 3D Printing at Hospitals and Medical Centers, or at the Point of Care. 3D Print Med..

[48] Morrison R.J., Kashlan K.N., Flanangan C.L., Wright J.K., Green G.E., Hollister S.J., Weatherwax K.J. (2015). Regulatory Considerations in the Design and Manufacturing of Implantable 3D-Printed Medical Devices. Clin. Transl. Sci..

[49] Rojek I., Dostatni E., Kopowski J., Macko M., Mikołajewski D. (2022). AI-Based Support System for Monitoring the Quality of a Product within Industry 4.0 Paradigm. Sensors.

[50] Carl A.-K., Hochmann D. (2022). Comparison of the Regulatory Requirements for Custom-Made Medical Devices Using 3D Printing in Europe, the United States, and Australia. Biomed. Technik. Biomed. Eng..

[51] Di Prima M., Coburn J., Hwang D., Kelly J., Khairuzzaman A., Ricles L. (2016). Additively Manufactured Medical Products—The FDA Perspective. 3D Print. Med..

[52] Wu S., Zeng J., Li H., Han C., Wu W., Zeng W., Tang L. (2023). A Review on the Full Chain Application of 3D Printing Technology in Precision Medicine. Processes.

[53] Pettersson A.B., Ballardini R.M., Mimler M., Li P., Salmi M., Minssen T., Gibson I., Mäkitie A. (2023). Legal Issues and Underexplored Data Protection in Medical 3D Printing: A Scoping Review. Front. Bioeng. Biotechnol..

[54] Ripley B., Levin D., Kelil T., Hermsen J.L., Kim S., Maki J.H., Wilson G.J. (2017). 3D Printing from MRI Data: Harnessing Strengths and Minimizing Weaknesses. J. Magn. Reson. Imaging JMRI.

[55] Parthasarathy J., Krishnamurthy R., Ostendorf A., Shinoka T., Krishnamurthy R. (2020). 3D Printing with MRI in Pediatric Applications. J. Magn. Reson. Imaging JMRI.

[56] Okkalidis N., Bliznakova K., Kolev N. (2022). A Filament 3D Printing Approach for CT-Compatible Bone Tissues Replication. Phys. Medica.

[57] Chopra S., Emran T.B. (2024). Advances in AI-Based Prosthetics Development: Editorial. Int. J. Surg..

[58] David S., Bačić B., Richter C., Mundt M. (2023). Editorial: Artificial Intelligence to Enhance Biomechanical Modelling. Front. Sports Act. Living.

[59] Siddiqui I.A., Littlefield N., Carlson L.A., Gong M., Chhabra A., Menezes Z., Mastorakos G.M., Thakar S.M., Abedian M., Lohse I. (2024). Fair AI-Powered Orthopedic Image Segmentation: Addressing Bias and Promoting Equitable Healthcare. Sci. Rep..

[60] Chia H.N., Wu B.M. (2015). Recent Advances in 3D Printing of Biomaterials. J. Biol. Eng..

[61] Abolhassani S., Fattahi R., Safshekan F., Saremi J., Hasanzadeh E. (2025). Advances in 4D Bioprinting: The Next Frontier in Regenerative Medicine and Tissue Engineering Applications. Adv. Healthc. Mater..

[62] Alexiou M.V., Tooulias A.I., Papadopoulos V.N., Tsioukas V., Suri J.S.B.T. (2022). Chapter 3-Three-Dimensional Bioprinting in Medical Surgery. 3D Printing: Applications in Medicine and Surgery Volume.

[63] Shi L., Wei W., Smith A., Abbasi G. (2024). Implementation and Evaluation of an EHR-Integrated Perpetual Inventory System in a Large Tertiary Hospital Oncology Pharmacy. Am. J. Health-Syst. Pharm..

[64] Osouli-Bostanabad K., Adibkia K. (2018). Made-on-Demand, Complex and Personalized 3D-Printed Drug Products. BioImpacts BI.

[65] Rezapour Sarabi M., Alseed M.M., Karagoz A.A., Tasoglu S. (2022). Machine Learning-Enabled Prediction of 3D-Printed Microneedle Features. Biosensors.

[66] Oladapo B.I., Bowoto O.K., Adebiyi V.A., Ikumapayi O.M. (2023). Net Zero on 3D Printing Filament Recycling: A Sustainable Analysis. Sci. Total Environ..

[67] Faruki A.A., Zane R.D., Wiler J.L. (2022). The Role of Academic Health Systems in Leading the “Third Wave” of Digital Health Innovation. JMIR Med. Educ..

[68] Mohd Noor M.N., Leow M.L., Lai W.H., Hon Y.K., Tiong L.L., Chern P.M. (2022). Research Landscape on 3D Printing Applications in Healthcare within Southeast Asian Countries: A Systematic Scoping Review Protocol. BMJ Open.

[69] Olatunji G., Osaghae O.W., Aderinto N. (2023). Exploring the Transformative Role of 3D Printing in Advancing Medical Education in Africa: A Review. Ann. Med. Surg. (2012).

[70] Rojek I., Mikołajewski D., Kempiński M., Galas K., Piszcz A. (2025). Emerging Applications of Machine Learning in 3D Printing. Appl. Sci..

[71] Jindal J.A., Lungren M.P., Shah N.H. (2024). Ensuring Useful Adoption of Generative Artificial Intelligence in Healthcare. J. Am. Med. Inform. Assoc..

[72] Zhang Z., Zhou X., Fang Y., Xiong Z., Zhang T. (2025). AI-Driven 3D Bioprinting for Regenerative Medicine: From Bench to Bedside. Bioact. Mater..

[73] Arbelaez Ossa L., Milford S.R., Rost M., Leist A.K., Shaw D.M., Elger B.S. (2024). AI Through Ethical Lenses: A Discourse Analysis of Guidelines for AI in Healthcare. Sci. Eng. Ethics.

[74] Kermavnar T., Shannon A., O’Sullivan K.J., McCarthy C., Dunne C.P., O’Sullivan L.W. (2021). Three-Dimensional Printing of Medical Devices Used Directly to Treat Patients: A Systematic Review. 3D Print. Addit. Manuf..

[75] Willemsen K., Nizak R., Noordmans H.J., Castelein R.M., Weinans H., Kruyt M.C. (2019). Challenges in the Design and Regulatory Approval of 3D-Printed Surgical Implants: A Two-Case Series. Lancet. Digit. Health.

[76] Martínez-Villaseñor L., Ponce H., Ponce H., Brieva J., Lozada-Flores O., Martínez-Villaseñor L., Moya-Albor E. (2024). Ethical Design Framework for Artificial Intelligence Healthcare Technologies BT. Data-Driven Innovation for Intelligent Technology: Perspectives and Applications in ICT.

[77] Cronin U.M., Cummins N.M., O’Sullivan A., O’Sullivan L. (2025). Perceived Barriers and Opportunities to the Use of 3D Printing in a Healthcare System with Low Adoption: A Semi-Structured Interview Study. HRB Open Res..

[78] Shoja M.M., Van de Ridder J.M.M., Rajput V. (2023). The Emerging Role of Generative Artificial Intelligence in Medical Education, Research, and Practice. Cureus.

[79] Yap Y.L., Tan Y.S.E., Tan H.K.J., Peh Z.K., Low X.Y., Yeong W.Y., Tan C.S.H., Laude A. (2017). 3D Printed Bio-Models for Medical Applications. Rapid Prototyp. J..

[80] Shiroorkar S., Shiroorkar P.N., Aman M., Gurlhosur S.S., Gangadhar S. (2025). Applications for 3D Printing in Healthcare System: Current Trends, Recent Developments and Future Prospects. Karnataka Med. J..

[81] Niha K., Surendiran B., Amutha S., Sharma N., Goje A.C., Chakrabarti A., Bruckstein A.M. (2024). Biomedical Data Management and Analytics in IOMT BT. Data Management, Analytics and Innovation.

[82] Kanthimathi T., Rathika N., Fathima A.J., S R.K., Srinivasan S., R T. (2024). Robotic 3D Printing for Customized Industrial Components: IoT and AI-Enabled Innovation. Proceedings of the 2024 14th International Conference on Cloud Computing, Data Science & Engineering (Confluence), Noida, India, 18–19 January 2024.

[83] Kang H.-W., Lee S.J., Ko I.K., Kengla C., Yoo J.J., Atala A. (2016). A 3D Bioprinting System to Produce Human-Scale Tissue Constructs with Structural Integrity. Nat. Biotechnol..

